# Impacts of Phosphogypsum, Soluble Fertilizer and Lime Amendment of Acid Soils on the Bioavailability of Phosphorus and Sulphur under Lucerne (*Medicago sativa*)

**DOI:** 10.3390/plants9070883

**Published:** 2020-07-13

**Authors:** Moussa Bouray, Jim Moir, Leo Condron, Niklas Lehto

**Affiliations:** 1 Department of Soil Science, Lincoln University, Lincoln 7647, Christchurch, New Zealand; Jim.Moir@lincoln.ac.nz (J.M.); Leo.Condron@lincoln.ac.nz (L.C.); Niklas.Lehto@lincoln.ac.nz (N.L.); 2 AgroBioSciences Program, Mohammed VI Polytechnic University (UM6P), Benguerir 43150, Morocco

**Keywords:** lucerne, acid soil, phosphogypsum, phosphorus, sulphur, exchangeable aluminum

## Abstract

Legumes play critical dual roles in grazed grassland ecosystems; providing nitrogen inputs and high-quality feed for grazing livestock. However, many species fail to persist in acidic, low fertility soils. A glasshouse study was conducted to investigate the response of lucerne (*Medicago sativa*) to phosphogypsum (PG), lime and soluble P + S fertilizer (PS) application to two soils. Phosphorus and sulphur were applied through either PG (0, 1, 3 and 9 t ha^−1^) or P + S fertilizer at equivalent rates to PG. Both PG and PS were applied with or without lime, which was applied at 2 t ha^−1^. Yield and nutrient uptake of the lucerne was measured, while the soil was analyzed for pH, Olsen P and exchangeable aluminum. Yield responses were significantly different between the two soils. Maximum yields and P and S uptakes were obtained under PG 9 t ha^−1^ combined with lime. Exchangeable Al decreased in both soils under 1 ha^−1^ of PG compared with the control. At the highest rate, Olsen P increased by 8 and 6 mg kg^−1^ for PG and by 6 and 11 mg kg^−1^ for PS compared with the control for Glenmore and Molesworth soils respectively. Phosphogypsum showed positive effects on P and S bioavailability.

## 1. Introduction

Soil acidity coupled with phytotoxic concentrations of soil exchangeable aluminum (Al) and low phosphorus (P) and very low sulphur (S) availability are among the major limitations to legume establishment and growth in New Zealand hill and high country farms [1,2,3]. Lucerne (Medicago sativa) is one of the most valuable forage legumes in New Zealand but is very sensitive to aluminum (Al) and manganese [4] toxicities that prevail in low pH [5,6] and P availability [3]. Hence the lime application is often an essential prerequisite to counteract soil acidity and enhance legume establishment and persistence in grasslands [7]. However, liming effectiveness is limited to the shallow top-soil layer and has a very limited effect on subsoil acidity in the short term [8] due to its low solubility and passive movement down the soil profile [9,10].

Most productive pasture legumes are adapted to highly fertile soils [1]. In contrast, soil fertility levels (plant-available P and S) are often low in many hill and high country soils in New Zealand [2] due to low fertilizer inputs [11] which is driven by the economics of fertilizer and lime application in these complicated topographical areas requiring aerial application [12]. As such, the sustainability of legume production in New Zealand hill and high-country pastures may depend on new alternative affordable sources of nutrients for the farmers.

Phosphogypsum (PG), a by-product of the phosphoric fertilizer industry, is produced when rock phosphate (fluorapatite) is digested with concentrated sulphuric acid according to the following chemical formula:

(1) $${\mathrm{Ca}}_{10}{\left({\mathrm{PO}}_{4}\right)}_{6}F_{2}+10{\;H}_{2}{\mathrm{SO}}_{4}+20{\;H}_{2}O\;↔\;6{\;H}_{3}{\mathrm{PO}}_{4}+10{\;\mathrm{CaSO}}_{4}.2{\;H}_{2}O+2\;\mathrm{HF}.$$

About 160 million tons of phosphogypsum are produced annually worldwide and it is mainly disposed of in large stockpiles or discharged in water bodies [13]. It contains predominantly sulphur and calcium oxide and small amounts of phosphorus [14]. It may contain small amounts of heavy metals and radioactive element impurities, whose concentrations depend on the composition of raw materials [15,16] and the processing method used [17]. Because of these impurities, the use of PG has been restricted in some markets, although these restrictions did not always have a proper scientific justification [13].

Phosphogypsum is used in agriculture all over the world, for example in Brazil, Spain, Australia, India, Pakistan, USA and Egypt [18,19,20], either as soil amendment under the category “Calcium sulphate” or as fertilizer [21]. Several benefits of PG application in agriculture have been reported worldwide for saline/sodic soils [22,23,24] or acidic soils [25,26,27,28]. However, research on PG application on acid soils has mostly focused on its effects in alleviating the toxic effects of high Al bioavailability or used in providing calcium for crops [27]. Studies examining PG effects on soil fertility in general and on P and S availability in acid soils (pH ≤ 5) for legumes are limited; to date, there has been no research on PG use in NZ grasslands.

The main objective of this study was to compare the effects of PG amendment, soluble fertilizer and lime on short-term lucerne yield and P and S uptake in two different acid soils under controlled environment conditions.

## 2. Results

### 2.1. Phosphogypsum Effects on Yield and Nutrient Uptake

**Figure 1:**
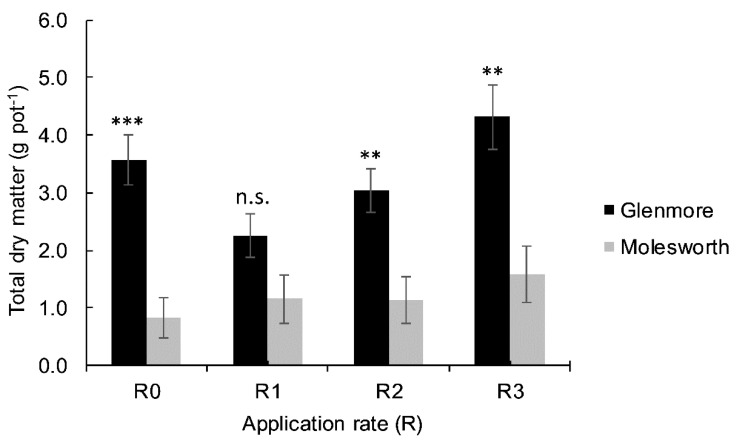
Total dry matter yield (g pot^−1^, n = 8) of lucerne (*Medicago sativa*) after six-month growth period under two different soils: Molesworth (MO) and Glenmore (GM), as affected by four rates of phosphogypsum (PG) across two lime treatments (0 and 2 t ha^−1^). Error bars indicate standard errors (± SEM, n = 8). Means of TDM for GM and MO per rate (R0 to R3) were separated using a two-sample *t*-test at 5%. Asterisks above bars indicate the level of significance in the difference between the two soils within each rate of PG (** *p* < 0.01, *** *p* < 0.001, n.s. not significant).

**Figure 2:**
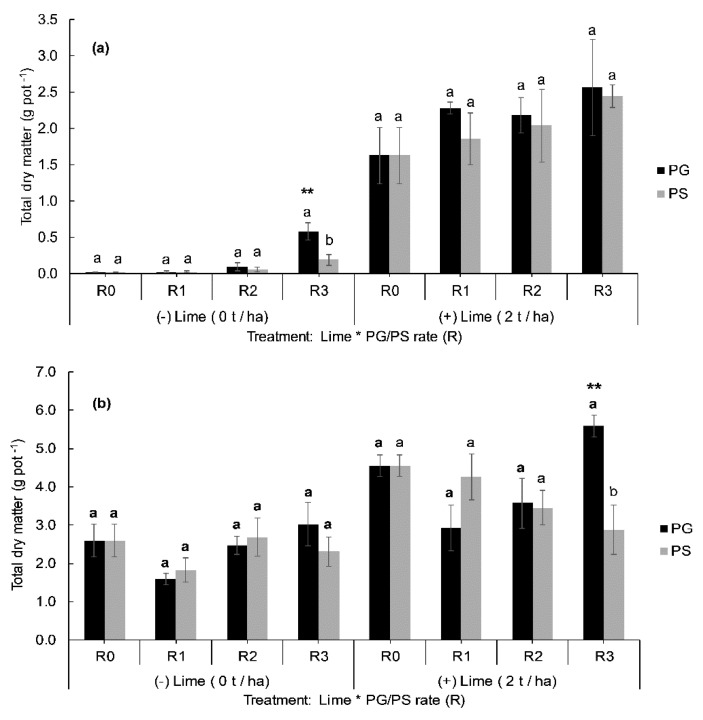
Comparison between the effects of phosphogypsum (PG) and soluble fertilizer (PS) on total dry matter yield (g pot^−1^, n = 4) of lucerne (*Medicago sativa*) after six month growth period under two different soils: Molesworth (**a**) and Glenmore (**b**) treated (+) or not (−) with lime. Error bars indicate standard errors (± SEM, n = 4). Means of TDM for PG and PS per rate (R0 to R3) were separated using a two-sample *t*-test at 5%, means indicated by the same lower-case letter per rate are not significantly different. Asterisks above bars indicate the application rate where TDM was significantly higher compared to the corresponding control under PG and PS separately, according to the Dunnett test at 5%. ** indicates a one-way ANOVA significance level (1%).

Plant response to soil inputs varied depending on the soil type and treatment type and rate (Figure 1 and Figure 2). Total dry matter (TDM) yields were different (*p* < 0.001) between the two soils, an average of 1.2 g and 3.2 g TDM yield per pot were recorded for Molesworth (MO) and Glenmore (GM) soils respectively, across all treatments. Likewise, the main effect of liming and phosphogypsum (PG) was significant on TDM yield per pot. However, the soluble fertilizer’s (PS) main effect was not significant (Table 1). Moreover, the effect of the interaction on the TDM yield was only significant for soil × PG (Figure 1). As such, the significance of the simple effect of PG was presented for GM and MO separately under the two lime rates (Figure 2).

At the highest tested rate, R3 = 9 t ha^−1^, the PG increased (*p* < 0.01) the TDM yield compared to the control (R0) in the unlimed MO soils (Figure 2a), whilst at the rates of (R1 = 1 ha^−1^) and (R2 = 3 t ha^−1^), the yields were lower and no significant effects were recorded compared to the control. Whereas, in unlimed GM soil, the PG (9 t ha^−1^) effect on the yield was not significant compared to the control unless combined with lime. The yields were greater in the presence of lime for both PG and PS application. Phosphogypsum at R3 (9 t ha^−1^) combined with lime (2 t ha^−1^) increased TDM yield in the order of 46% and 77% compared with PG (9 t ha^−1^) alone for GM and MO respectively. Similarly, soluble fertilizer applied at the same rates of S and P and combined with lime (R3 + Lime), increased the yield by 20% and 91% compared to PS (R3) alone for MO and GM respectively.

The comparison between PG and PS in terms of TDM yields generated per rate under the two investigated soils and lime rates are shown in Figure 2. Trends in dry matter yields were relatively similar for PG and soluble fertilizer (PS) treatments in MO soil (Figure 2a); the yields increased proportionally to the application rate. However, where lime was added, an opposite trend was observed between PG and PS for GM soil (Figure 2b). In most cases, the average TDM yield produced per rate were not different (*p* > 0.05) between PG and PS regardless of soil and lime effects, except for R3 under unlimed MO and R3 under limed GM where PG effect was significantly (*p* < 0.01) higher compared to PS.

For the MO soil, most of the treatments without lime did not result in enough shoot dry matter for herbage analysis; the quantities harvested were less than 0.2 g which is the minimum required shoot weight for nutrients analysis through digestion solution, thus data of P and S uptakes were not determined. Shoots of the plants grown on limed GM soil showed higher P and S uptakes than those grown on limed MO soil. Significant treatment effects were recorded for the two soils regarding S uptake (Table 2). The highest S uptakes were found under PS (R3) and PG (R3) both combined with lime irrespective of soil type, though PG (R3) either combined with lime or not significantly enhanced S uptake compared with the control under GM soil. For P, the highest uptakes were recorded under PG (R3) combined with lime regardless of soil type. For example, under limed GM soil, PG (R3) increased (*p* < 0.05) P uptake by 35% compared to PS (R3) and by 21% compared with the control (R0). However, without lime addition, PG’s effect on nutrient uptake, particularly P, was similar to PS.

The multiple linear regression analysis results are presented in Table 3. The data of PG and PS treatments were compiled to conduct this analysis (n = 128). The comparison of standardized coefficients for the three considered variables (pH, exchangeable Al and Olsen P) revealed that pH was the most important factor in impacting the TDM yield followed by Olsen P, then exchangeable aluminum. The interaction between these three predictors was not significant, therefore excluded from the model. The regression equation is presented below:

(2) $$\mathrm{TDM}\;\mathrm{yield}=-13.20+2.89{\;\mathrm{pH}}_{\mathrm{CaCl}2}+0.042\;\mathrm{Exchangeable}\;\mathrm{Al}+0.082\;\mathrm{Olsen}\;P$$

(n = 128, *p* < 0.001, Adjusted R^2^ = 25%)

### 2.2. Phosphogypsum Effects on Soil pH, Olsen P and Exchangeable Aluminum

Soil pH and exchangeable Al concentrations in both soils changed during the six-month growth period (Table 4 and Table 5). There were decreases in both pH (H_2_O) and pH (CaCl_2_) between the highest and lowest PG application rates in the GM soil and for the pH (H_2_O) in the MO soil, while the pH (CaCl_2_) in the MO soil was relatively stable. Adding lime increased the pH of both soils and while the PG treatments mostly negated this effect at the highest rate, the lime generally reduced the acidifying effect of increasing PG application rate. When lime was applied, a significant decrease in pH was seen in the pH (H_2_O) of both soils between the highest and lowest PG application rates. Another notable exception was the pH (CaCl_2_) in the MO soil, where the effect of the lime was comparatively similar across the four levels of PG. The PS treatments did not significantly change the pH (H_2_O) of the soils. Further, an increase (*p* < 0.05) in pH (CaCl_2_) was observed at the highest rate (R3) compared with the corresponding controls for limed GM and unlimed MO soils.

Exchangeable aluminum concentrations under different treatments in both soils exceeded the toxicity threshold of 3 mg kg^−1^ [3] for most grassland legume species. However, a substantial decrease of exchangeable Al content was observed for some treatments. For example, 5.9 and 7.5 mg kg^−1^ decrease of exchangeable Al concentration in GM and MO respectively were found under limed control (R0) compared with the unlimed control. A decrease of 5.3 and 2.4 mg kg^−1^ of exchangeable Al was recorded in unlimed GM and MO soils respectively at R1 = 1 t ha^−1^ of PG compared with the corresponding controls, also a reduction of 5.7 mg of exchangeable aluminum per kg of unlimed MO soil at R2 (3 t PG ha^−1^) compared with the corresponding control was observed. Whereas, at R3 (9 t PG ha^−1^) the soil exchangeable aluminum increased in both soils. The same effect has been observed for soluble fertilizer PS (R3) in both soils but PS was less effective in reducing soil exchangeable Al at low rates (R1 and R2) compared to PG; a decrease of 2.9 mg kg^−1^ was observed for PS (R1) in the unlimed GM soil only. The exchangeable Al concentrations were relatively higher across all PS treatments compared to PG treatment in the absence of lime.

**Figure 3:**
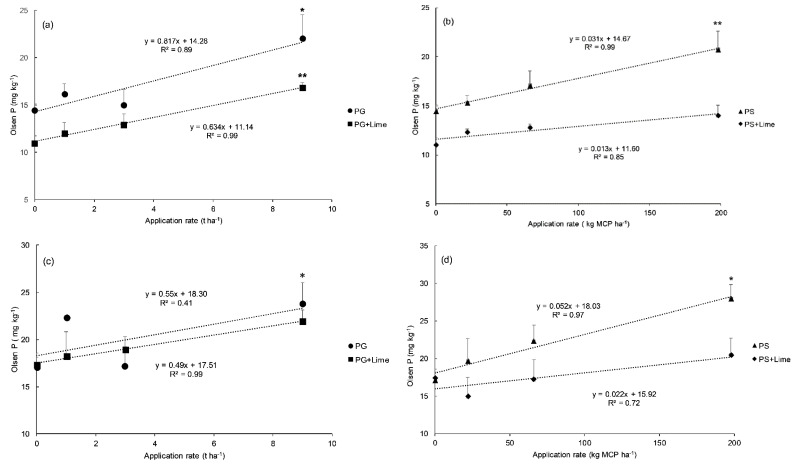
The relationship between mean Olsen P (mg kg^−1^, n = 4) and application rate of phosphogypsum (PG, t ha^−1^)) and soluble fertilizer (PS, kg of MCP ha^−1^) combined or not with lime under Molesworth (**a**,**b**) and Glenmore (**c**,**d**) soils, separately. Error bars indicate upper standard errors (SEM, n = 4). MCP: mono-calcium phosphate (CaHPO_4_). Asterisks indicate the application rates which are significantly different from the control (0 tons or 0 kg ha^−1^), according to the Dunnett test at 5%. * and ** indicate a one-way ANOVA significance level of 5 and 1%.

The relationships between PG and PS treatments and soil Olsen P are presented in Figure 3. The Olsen P measured in both soils increased (*p* < 0.05) in the presence of PG with a maximum recorded at 9 t PG ha-1 where Olsen P increase by 8 and 7 mg kg^−1^ compared with the control (0 t ha^−1^) for MO and GM soils respectively. Similarly, PS increased (*p* < 0.05) Olsen P by 6 and 11 mg kg^−1^ at the highest rate (198 kg MCP ha^−1^) compared with the control (0 kg MCP ha^−1^) for MO and GM soils respectively. The average Olsen P across all treatments of PG or PS was higher (*p* < 0.05) in GM soil compared to MO soil for both 0 and 2 t lime ha^−1^ (Table 6). The average Olsen P across all PG rates decreased under liming in both soils but to a lesser extent compared to PS. For example, in limed GM soil, the average Olsen P for PG decreased by 0.9 mg kg^−1^ compared to the unlimed GM. However, for PS the decrease was 5 times higher than that of PG. A similar trend was observed in the limed MO soil compared to unlimed MO, though the difference between PG and PS is not as large as in GM soil.

## 3. Discussion

### 3.1. Phosphogypsum Effects on Yield and Nutrient Uptake

The difference in TDM yields produced under MO and GM soils regardless of treatment type can be attributed to the initial soil fertility. Low soil pH coupled with high exchangeable Al in the MO soil prevented seedlings from germinating and establishing (TDM = 0 g pot^−1^ for control), thus depressing the overall average TDM in that soil compared with GM. Despite showing a decrease in exchangeable Al at 1 and 3 t ha^−1^, PG effects on TDM yields were less efficient than lime alone even at high application rates independently of soil type. These findings support the evidence that reducing exchangeable Al without improving soil pH will not be sufficient for legumes to persist. Therefore, lime was more efficient as it significantly increased soil pH while reducing exchangeable aluminum, which was not the case for PG. However, the mixture PG + Lime enhanced dry matter yield better than lime alone due to the supply of P and S through PG application. These two elements are considered the most limiting edaphic requirement to legumes in NZ hill and high country farms [1,29] which is consistent with the highest DM yields being observed when PG + Lime or PS + Lime were applied to MO soil. However, under GM soil TDM yield response to PS and PG did not support this hypothesis, except for PG at the highest rate where a significant increase in the yield was observed compared to the control under liming conditions.

The response to PG + Lime or PS + Lime was more pronounced for the MO soil compared to the GM soil, presumably because of the low initial P and S content of MO soil. The SO_4_^2−^-S level of the GM soil was above 10–12 mg S kg^−1^, which is the range for near-maximum pasture on hill and high country farms in NZ [30]. For the MO soil, SO_4_^2−^-S level was below that range and its initial Olsen P was 5 units lower than GM. Additionally, the poor response to PS and PG in the unlimed GM soil compared to unlimed MO soil in terms of TDM yield could also be due to the exhibited higher Al bioavailability. For example, the drop in TDM yield observed at PS (R3) under unlimed GM soil was coincided with a significantly higher exchangeable Al concentration compared to the rest of the PS treatments.

The improvement of TDM yield under limed GM at PG (R3) against PS (R3) is supported by the significantly higher P uptake and higher S uptake for PG (R3) compared to PS (R3). This was in agreement with the difference in Olsen P concentration between the two treatments (∆Olsen P = 1.5 mg kg^−1^) even though PS (R3) has greatly increased Olsen P compared to PG (R3) in the absence of lime (∆Olsen P = 4 mg kg^−1^) for the same soil. These findings suggest that the depressive effect of lime on P availability is higher for PS than PG. Moreover, at R3 (9 t ha^−1^), PG supplied large amounts of Ca which were found to decrease Al activity in soil solution when Ca^2+^/Al^3+^ ratio is high even if exchangeable Al is high [31], therefore alleviating its deleterious effects on roots [32,33].

The fact that P uptakes were not significantly affected by P supply for PG and PS treatments in the absence of lime compared with the control (R0), gives insight that this was likely related to other factors, probably soil pH. This explanation is strongly supported by higher P uptakes measured under Lime + PG and Lime + PS where pH is significantly higher. Similar findings were recently found by Otieno et al. [34] in acid soils of western Kenya. Additionally, the combination PG + L has been reported to stimulate soil microbial activity [35,36] and this could have improved phosphorus bioavailability.

The continuous increase in shoot uptaken S per pot with PG and PS rate increase indicates that lucerne was still S-uptake responsive to S supply even at high rates and that PG can be an alternative source for S fertilization as it performed almost identically to soluble fertilizer in both soils. The sulphur concentration of shoots under different PG and PS rates exceeded the optimum range of 0.18–0.22% S, suggested by Craighead and Metherell [37] for NZ high and hill country farms.

Lime addition increased S uptake under both PG and PS treatments. This was likely due to the mobilization of the adsorbed SO_4_^2−^ at low pHs [38,39]. The effect of lime is usually attributed to the competition between OH^−^ and SO_4_^2−^ on adsorption sites on Fe and Al hydrous oxides and P compounds may also compete for adsorption sites as they become more soluble at higher pHs [40]. Furthermore, the enhancement of root growth following lime application could also explain the greater uptakes of P and S with increased soil pH (Figure S1).

We can conclude from the negative linear relationships exhibited between shoot dry matter yields and the shoot concentration of P and S (Table 7), that P and S supply may not be limiting the yield in GM soil. This explanation is supported by the observed mean nutrient concentrations in the plant shoots, which are in ‘’adequate’’ range according to Craighead and Metherell; Morton et al.; Venter et al. [37,41,42]. This hypothesis is also in line with the TDM yield data in GM soil where no significant differences were found between the control and treated soils with P and S either through PS or PG, except PG (R3 + Lime). Conversely, the decrease of P and S content of shoots with increased yield could be due to the ‘’ dilution effect’’ associated with the extra dry matter production [43].

The correlation matrix showed weak relationships between nutrients concentrations and shoot yield under MO soil. However, the mean P concentrations across all treatments were below deficiency values. This indicates that the uptake of P is not solely dependent on its availability in this soil but influenced by other factors, most likely soil pH. Although no strong correlation was found between shoot yield and shoot S concentrations, an increase in S concentration was recorded when sulphur was supplied through PG and PS compared to the control. This could be explained by the luxury consumption of S by lucerne [42], which means that not all the S removed by the crop is essential for plant growth.

### 3.2. Phosphogypsum Effects on Soil pH, Olsen P and Exchangeable Aluminum

The lower soil pH (H_2_O) resulting from phosphogypsum addition was consistent with the findings of Nayak et al.; Lee et al.; Jarak et al. [24,35,44]. The PG used in this study had a high calcium content which likely displaced Al^3+^ and H^+^ on cation exchange sites into the soil solution resulting in low pH, this view is supported by the measured pH (H_2_O) under PS treatments, where Ca supply was largely lower compared to PG, which resulted in a relatively stable pH (H_2_O). Besides, the low pH (H_2_O, 3.5) of this PG product likely contributed to acidifying the soil. On the other hand, Smith et al. [45] found that the pH of surface amended soil with 2.5 t ha^−1^ of PG was unchanged.

The pH (CaCl_2_) results in this study showed an opposite trend to pH (H_2_O), especially for unlimed MO soil as its pH (CaCl_2_) increased slightly with PG. This confirmed what was reported recently about the increase of soil pH at depths of 0 to 5 cm by Crusciol et al. [27], after the surface application of phosphogypsum. Further, this result corroborates early reports by Toma et al. [4], who found that gypsum application decreased pH (H_2_O) while pH (CaCl_2_) did not. This could be explained by pH (H_2_O) overshadowing the liming effect of phosphogypsum due to the salt effect, whereas measuring pH in 0.01 M CaCl_2_ kept constant the effect of salt on the hydrolysis of Al forms releasing H^+^ protons. Moreover, in a pot experiment conducted by Edmeades et al. [7] where they recorded the same behavior between pH (KCl) and pH (H_2_O), this was claimed to be related to the decrease of ionic strength of the solution after dilution with H_2_O. Therefore, in glasshouse studies where soil volume is limited promoting high ionic strength conditions, the interpretation of pH (H_2_O) must be done carefully. Similarly, CaCl_2_ extracted pH increased significantly under PS for unlimed MO and limed GM. The observed rise in pH (CaCl_2_) following PS and PG application to the soil can also be ascribed to ligand exchange, whereby the supplied SO_4_^2−^ replaces OH^−^ [46,47]. So, considering these factors, we can state that the PG and PS effect on soil pH likely depends on the balance between Ca^2+^ and SO_4_^2−^ reactions.

The observed reduction in soil Al concentrations under soils treated with 1 t ha^−1^ of PG agrees with Crusciol et al. [27]. This effect can be explained by the association of the Al^3+^ ions with SO_4_^2−^ and F^−^ forming ionic pairs AlSO^4+^, AlF_2_^+^, AlF^2+^ and AlF_3_^0^ [48]. The absence of PG restriction effects on exchangeable Al at 9 t ha^−1^ for both MO and GM soils was probably due to the higher ionic strength in the soil solution. This would favor ionic exchange reactions to the detriment of adsorption and precipitation reactions. Hence, the ionic exchange of Ca^2+^ would have increased Al^3+^ in the solution and dominated the ligand exchange reactions. Additionally, at PG (9 t ha^−1^) the pH (H_2_O) has dropped by 0.4 and 0.34 units compared to the control in unlimed GM and MO soils respectively. This triggered an increase of exchangeable Al in GM and MO soils. This inverse relationship between pH and exchangeable Al in New Zealand soils has been confirmed by several researchers [8,49,50,51,52,53,54,55]. However, the observed increase in exchangeable Al under PS treatments was not related to soil pH which was unchanged. This agrees with the findings of Horsnell [56], in a glasshouse experiment, where those workers found that neutral salts (K_2_SO_4_ or CaSO_4_) in the presence of calcium phosphate increased aluminum concentrations in soil solution.

The difference between the influence of PG (R1 = 1 t ha^−1^) and PG (R3 = 9 t ha^−1^) in the absence of lime on Olsen P was larger (6 units) for MO soil, while under GM soil it was small (only 1 unit). This has probably resulted from a sharp decrease in GM soil pH when 9 t ha^−1^ of PG was applied, which would have consequently increased P adsorption [57] on oxide surfaces. This explanation is also supported by the high exchangeable Al content measured in unlimed GM under PG (9 t ha^−1^), which exceeded that of PG (1 t ha^−1^) by 14.6 mg kg^−1^ of soil.

The effect of PG on soil P availability is comparatively similar to that of soluble fertilizer used in this study as a standard source of P and S. This indicates the high solubility of total P contained in PG materials and its ability to be easily released into the soil and therefore be available to the plants in the same manner as soluble fertilizers. Phosphogypsum amendment could also have improved the microbial activity and population in the soil [24,35,36,58] resulting in a higher P solubility.

Lime application decreased Olsen P for both soils regardless of treatment type. Our results are in line with studies done by other workers on acid soils [59,60]. This decline in soil P under lime application can be due to the formation of Ca-P precipitates [61]. Moreover, when pH is increased, the proportion of absorbable P species increases such as the divalent phosphate (HPO_4_^2−^) [62]. The formation of insoluble hydroxyl-Al species following lime can also be highly active adsorption surfaces for phosphate [63]. Haynes and Ludecke [64] reported an increase in Al-bound P fraction under liming. Moreover, the stability of hydroxyl-Al-P complexes has been reported to be high around pH 5 [65]. However, the decrease in P availability following liming is not supported by plant shoot P uptake in this experiment, which showed a significant increase when lime was applied. Alternative soil P tests under liming conditions are recommended as the decline of Olsen P could be due to an artifact in the Olsen procedure which uses high pH extractant (pH 8) favoring the Ca-P precipitation [60].

## 4. Materials and Methods

### 4.1. Soil Characteristics

Two acid soils with different chemical and physical properties were used (Table 8). They were collected from two different sites and are known to be phosphorus-deficient and have high bioavailable Al concentrations. The “GM” soil was sampled from Glenmore station, located on the southern banks of Lake Tekapo, central Canterbury, while the “MO” soil was collected from Molesworth station, in the Marlborough region. The two soils are classified as Dystrudepts [66] or Brown soils (NZ soil classification after [67]). Upon collection (0–15 cm), plant material and stones were removed. The soils were air-dried and sieved (4 mm mesh).

### 4.2. Experimental Design and Treatments

The sieved soils were subjected to one of four treatments. In PG treatment, four rates of phosphogypsum: 0, 1, 3 and 9 t ha^−1^ (5.4, 16.2 and 48.6 kg P ha^−1^ and, 113, 339 and 1017 kg of S ha^−1^ respectively) were applied. In the soluble fertilizer (PS) treatment, P and S were applied at four rates to match the amount of the nutrients in the PG treatment: P was supplied as 0, 22, 66 and 198 kg of monocalcium phosphate (CaHPO_4_) ha^−1^, while S was supplied as 0, 0.5, 1.5 and 4.5 t ha^−1^ of Sodium sulphate (Na_2_SO_4_). The rates of PG and PS were gradually increased to achieve an optimum Olsen P range of 25–30 mg kg^−1^ for lucerne [80,81] at the highest rate. The chemical composition of PG and soluble fertilizers used in this study is presented in Table 9.

The four rates of PG and PS are reported in the text, tables and figures as R0, R1, R2 and R3 respectively and each rate supplies the same amount of P and S for both PG and PS. The control treatment corresponds to rate 0 (R0) where no P and S inputs were supplied.

Both PG and soluble fertilizer (PS) treatments were applied alone (without lime: 0 t ha^−1^) but were also applied in combination with lime (2 t CaCO_3_ ha^−1^, lab-grade lime). This experiment was a 4 × 2 × 2 factorial design with 4 rates of PG or PS separately, two soils and two lime rates (0 and 2 t ha^−1^). Four replicates were used for each treatment level, giving a total of 112 pots. The lime treatment was included to increase soil pH and thus facilitate seedling emergence and plant establishment and to test for possible interactions with PG and PS. The lime rate of 2 t ha^−1^ has been used based on the findings of a pot experiment [82]. It has been reported to be an optimum lime rate for lucerne yielding under the same soil types investigated in this study.

Lime, PG and soluble fertilizer treatments were thoroughly mixed with 200 g of air-dried soil. Basal potassium (K) was also mixed with the soil (300 kg of K ha^−1^ as KCl). The treated soils were deployed in 250 mL plastic plant pots (66 mm diameter × 75 mm height) and distributed in a complete randomized block design on a table at Lincoln University (Lincoln, NZ) glasshouse facilities. The daily average temperature for the experiment period was 18 °C. A small pot size was used to create a rhizosphere environment where nutrient uptake could be enhanced and to speed up soil nutrient cycling enabling to see treatment effect in short period of time. However, this could negatively impact plant growth.

Lucerne (*M. sativa*, cv. Grasslands Kaituna) seeds were directly sown into the pots. After germination, the plants in each pot were thinned to 3 seedlings per pot and grown for six months, between March 16th to September 23rd, 2018. The pots were inoculated with a commercial (diluted peat culture) rhizobia strain, Group AL (New-Edge Microbials Pty. Ltd., Albury, Australia) 30 days post-germination to insure that an active rhizobia population is present in the soil. Throughout the growth period, soil moisture was monitored using high-frequency capacitance volumetric water content sensors (Decagon 5TM, Decagon Devices LTD, Pullman, Washington, USA) installed within the soil and maintained at 22–25% (v/v) by an automated dripper irrigation system.

### 4.3. Plant and Soil Sampling and Analysis

Lucerne shoots were harvested four times during the experiment period by cutting 2 cm above the crown of each plant, oven-dried at 70 °C for 48 h, weighed, finely ground and bulked on an individual pot basis. Therefore, shoot nutrient uptake data shown in this study represents the average of the four harvests and shoot DM yields data represents the sum of the four harvests per pot. At the end of the experiment, the roots were harvested, whereupon they were carefully cleaned using deionized water, dried at 70 °C for 48 h and then weighed. The soils were then collected and air-dried at 30 °C for 7 days. After drying the soil was sieved (2 mm) and stored at room temperature in polyethylene bags to await for analysis.

The chemical characteristics of the soils were determined using standard methods (Table 8). Soil pH (1:2.5 soil: water ratio) was measured using both deionized water and 0.01 M CaCl_2_. Bioavailable soil P (Olsen P) was extracted using 0.5 M sodium bicarbonate and was analyzed in a discrete wet chemistry analyzer (Smartchem TM 200, AMS Alliance, Paris, France). Exchangeable aluminum was extracted using 0.02 M CaCl_2_ (1:4 soil: water ratio) and analyzed using Inductively Coupled Plasma Optical Emission Spectrophotometry (ICP-OES: Varian 720-ES ICP-OES, Varian, Melbourne, Australia).

Herbage samples underwent acid digestion (Nitric acid (HNO_3_ 69%)-Hydrogen Peroxide (H_2_O_2_ 30%), 1:1 v/v) using a microwave digester (CEM MARS XpressTM, CEM Corp., Matthews, North Carolina, USA) [83]. The digest solution was analyzed for total P and S using Inductively Coupled Plasma Optical Emission Spectrophotometer (ICP-OES: Varian 720-ES ICP-OES, Varian, Melbourne, Australia).

### 4.4. Statistical Analysis

The data were analyzed at the end of the experiment and were subjected to analysis of variance (ANOVA) using Minitab^®^ statistical software version 8 (Minitab, Inc., State College, Pennsylvania, USA). The phosphogypsum or soluble fertilizer treatments, lime treatments and soil types were considered as fixed factors. Three-way ANOVA was carried out to test the significance of the main effect of each factor and to identify any significant interactions between them, it was carried out for (PG, lime and soil types) and (PS, lime and soil types) separately. One-way-ANOVA was used to test the effect of treatment levels on soil parameters (Olsen P, pH and exchangeable Al) and plant parameters (yield and uptakes). Where differences between the means were significant (*p* < 0.05), the Dunnett test (α = 5%) was used to compare treatment levels to the control level. The comparison between PS and PG effect per rate on TDM yield was performed using a two-sample *t*-test at 5%. The effects were considered to be significant when *p* ≤ 0.05. A correlation matrix was developed using the Pearson method to establish the relationships between P and S concentrations in the shoots and shoot DM yields. A simple linear regression was used to study the relationship between PG and PS application rates and soil Olsen P. A multiple linear regression (backward elimination) was used to determine the most important soil variable (pH, Olsen P or exchangeable Al) in impacting TDM yield. The variables were standardized by subtracting the mean then divide by standard deviation.

## 5. Conclusions

Phosphogypsum application on acid soils showed positive effects on soil P availability, P and S uptakes and consequently on lucerne biomass production. However, the effects of PG on soil acidity depends on soil properties. Its magnitude of pH-neutralizing effects does not support PG to be used as a lime substitute but rather as a fertilizer supplement. The responses to PG would not be maximized without being necessarily combined with a pH ameliorant such as lime in our case. The strategy of blending materials might be feasible for acid soils and can be a real solution for the improvement of lime solubility and therefore its reaction time and movement down to the sub-soil layers. However, there is a lack of evidence about it, hence further studies are required in this aspect. Phosphogypsum has decreased exchangeable Al at low rates, this warrants further investigations are necessary to evaluate the effects of PG on Al species. Further studies are also required to identify Al toxicity thresholds for legumes. The Ca effect on Al activity and phytotoxicity could also support the use of Ca-rich materials such as PG on acid soils.

The sustainable utilization of PG in agriculture necessitates long-term experiments focusing not only on fertilizing effects of PG but paralleled with an environmental impact assessment. Also, the assessment of PG effects on nutritional imbalances especially at high application rates is needed.

Soluble fertilizer used in this study (MCP + NaSO_4_), if used without lime, may not be the most effective for the establishment and growth of lucerne on acid soils with high native exchangeable aluminum levels. The increased Al concentrations following the combination of MCP and NaSO_4_ requires further investigation.

## Figures and Tables

**Table 1:** Summary of the analyses of variance to evaluate the effect of soil type, lime and phosphogypsum (PG) or soluble fertilizer (PG) and their interactions on total dry matter yield of lucerne (TDM, g pot^−1^).

Factors	Phosphogypsum (PG)	Soluble Fertilizer (PS)
Soil (S)	***	***
Lime (L)	***	***
Rate (R)	***	n.s.
S * L	n.s.	n.s.
S * R	**	n.s
L * R	n.s.	n.s
S * L * R	n.s.	n.s

Asterisks indicate significant effect levels (* *p* < 0.05, ** *p* < 0.01, *** *p* < 0.001), n.s. not significant.

**Table 2:** The effects of phosphogypsum (PG), soluble fertilizer (PS) and lime on lucerne (*Medicago sativa*) shoot P and S uptakes from Glenmore and Molesworth soils after a six-month plant growth period. Within rows, means followed by the same lower-case letter are not significantly different (Dunnett test at 5%, R0 = control). Within columns, means were compared using a two-sample *t*-test at 5%.

		P Uptake (mg pot^−1^)					S Uptake (mg pot^−1^)				
		R0	R1	R2	R3	*p* Value ^‡^	R0	R1	R2	R3	*p* Value
Glenmore (GM)											
(−) Lime	Phosphogypsum	3.76	2.31	3.11	3.87	0.084 n.s.	4.55a	4.31a	5.96b	8.32b	<0.001 ***
	Soluble fertilizer	3.76	2.79	3.78	2.87	n.s.	4.55	4.57	5.71	7.69	n.s.
	*p* value ^†^	n.s.	n.s.	n.s.	n.s.		n.s.	n.s.	n.s.	n.s.	
(+) Lime	Phosphogypsum	4.44	3.73	4.61	5.65	n.s.	5.92a	5.79a	7.72a	9.77b	<0.001 ***
	Soluble fertilizer	4.44	3.89	4.15	3.65	n.s.	5.92	6.77	7.13	8.36	0.067 n.s.
	*p* value	n.s.	n.s.	n.s.	0.043 *		n.s.	n.s.	n.s.	n.s.	
Molesworth (MO)											
(−) Lime	Phosphogypsum	n.d.	n.d.	n.d.	0.50	N.A.	n.d.	n.d.	n.d.	1.33	N.A.
	Soluble fertilizer	n.d.	n.d.	n.d.	n.d.	N.A.	n.d.	n.d.	n.d.	n.d.	N.A.
	*p* value	N.A.	N.A.	N.A.	N.A.		N.A.	N.A.	N.A.	N.A.	
(+) Lime	Phosphogypsum	1.43	2.08	2.26	2.52	0.053 n.s.	1.97a	3.46a	3.86b	5.36b	0.001 **
	Soluble fertilizer	1.43	1.68	1.81	2.20	n.s.	1.97a	3.02a	3.63a	5.67b	0.002 **
	*p* value	n.s.	n.s.	n.s.	n.s.		n.s.	n.s.	n.s.	n.s.	

Asterisks indicate significant effect levels (* *p* < 0.05, ** *p* < 0.01, *** *p* < 0.001). N.A. not applicable, n.d. not determined, n.s. not significant, ^‡^ One-way ANOVA at 5%, ^†^ two-sample *t*-test at 5%.

**Table 3:** Multiple linear analysis regression results (coded coefficients).

	Standardized Coefficients	SE Coefficients	*t* Value	*p* Value	VIF
Constant	2.15	0.13	16.63	*p* < 0.001	
pH (CaCl_2_)	0.70	0.14	5.08	*p* < 0.001	1.14
Exchangeable Al	0.31	0.16	2.01	0.047	1.45
Olsen P	0.40	0.15	2.55	0.012	1.43

VIF variance inflation factor, SE standard error.

**Table 4:** Effects of phosphogypsum and soluble fertilizer on soil pH (water and CaCl_2_) and exchangeable aluminum in both Glenmore and Molesworth soils under no lime application, after six months plant growth period. Within rows, means followed by the same lower-case letter are not significantly different (Dunnett test at 5%, R0 = control). Within columns, means were compared using a two-sample *t*-test at 5%.

	Phosphogypsum (PG)					Soluble Fertilizer (PS)				
	R0	R1	R2	R3	*p* Value ^‡^	R0	R1	R2	R3	*p* Value
pH water										
Glenmore (GM)	4.84a	4.82a	4.63a	4.42b	0.037 *	4.84	5.03	5.00	4.86	n.s.
Molesworth (MO)	4.83	4.66	4.59	4.49	n.s	4.83	4.81	4.80	4.82	n.s.
*p* value ^†^	n.s.	n.s	n.s.	n.s.		n.s.	0.041 *	n.s.	n.s.	
pH CaCl_2_										
Glenmore (GM)	4.47	4.39	4.31	4.23	n.s.	4.47	4.38	4.41	4.46	n.s.
Molesworth (MO)	4.16	4.24	4.30	4.31	n.s.	4.16a	4.33b	4.30a	4.43b	0.006 **
*p* value	n.s.	0.040 *	n.s.	n.s.		n.s.	n.s.	n.s.	n.s.	
Exchangeable Al (mg kg^−1^)										
Glenmore (GM)	22.6	17.3	26.6	31.9	n.s.	22.6a	19.7a	29.7b	31.8b	0.037 *
Molesworth (MO)	20.9	18.5	15.2	21.0	n.s.	20.9	21.5	20.1	20.7	n.s.
*p* value	n.s.	n.s.	n.s.	n.s.		n.s.	n.s.	n.s.	n.s.	

Asterisks indicate significant effect levels (* *p* < 0.05, ** *p* < 0.01, n.s. not significant). R0 to R3 indicates the four rates of PG (0, 1, 3 and 9 t ha^−1^) and their equivalence for PS. ^‡^ One-way ANOVA at 5%; ^†^ two-sample *t*-test at 5%.

**Table 5:** Effects of phosphogypsum and soluble fertilizer on pH (water and CaCl_2_) and exchangeable aluminum in Glenmore and Molesworth soils under liming (2 t ha^−1^) conditions. Within rows, means followed by the same lower-case letter are not significantly different (Dunnett test at 5%, R0 = control). Within columns, means were compared using a two-sample *t*-test at 5%.

	Phosphogypsum (PG)					Soluble Fertilizer (PG)				
	R0	R1	R2	R3	*p* Value ^‡^	R0	R1	R2	R3	*p* Value
pH water										
Glenmore (GM)	5.26a	5.16a	5.00b	4.66b	<0.001 ***	5.26	5.27	5.27	5.20	n.s.
Molesworth (MO)	5.35a	5.23a	5.03b	4.82b	<0.001 ***	5.35	5.33	5.33	5.32	n.s.
*p* value ^†^	n.s.	n.s.	n.s.	0.042 *		n.s.	n.s.	0.018 *	n.s.	
pH CaCl_2_										
Glenmore (GM)	4.64a	4.74a	4.73a	4.52b	0.011 *	4.64a	4.75a	4.80a	4.86b	0.043 *
Molesworth (MO)	4.73	4.74	4.74	4.65	n.s.	4.73	4.76	4.82	4.88	n.s.
*p* value	n.s.	n.s.	n.s.	0.045 *		n.s.	n.s.	n.s.	n.s.	
Exchangeable Al (mg kg^−1^)										
Glenmore (GM)	16.7a	16.8a	20.6a	30.4b	0.004 **	16.7	16.3	19.5	22.0	n.s.
Molesworth (MO)	13.4	10.9	12.9	20.2	n.s.	13.4	15.7	13.2	14.3	n.s.
*p* value	n.s.	0.026 *	n.s.	n.s.		n.s.	n.s.	n.s.	n.s.	

Asterisks indicate significant effect levels (* *p* < 0.05, ** *p* < 0.01, *** *p* < 0.001, n.s. not significant). R0 to R3 indicates the four rates of PG (0, 1, 3 and 9 t ha^−1^) and their equivalence for PS. ^‡^ One-way ANOVA at 5%, ^†^ two-sample *t*-test at 5%.

**Table 6:** Average soil Olsen P (mg kg^−1^ ± SEM) measured after 6 months of phosphogypsum (PG), soluble fertilizer (PS) and lime application to two different soils (Glenmore and Molesworth). Within columns, means followed by the same lower-case letter are not significantly different (Dunnett test at 5%). Within rows, means were compared using a two-sample *t*-test at 5%.

	(−) Lime (0 t ha^−1^)			(+) Lime (2 t ha^−1^)		
	Glenmore	Molesworth	*p* Value ^‡^	Glenmore	Molesworth	*p* Value
Control	17.1 ± 1.51a	14.5 ± 0.67a	n.s.	17.3 ± 0.80a	11.0 ± 0.73a	0.002 **
Phosphogypsum	20.0 ± 1.10a	16.9 ± 1.06a	0.051 n.s.	19.1 ± 0.86a	13.2 ± 0.70b	<0.001 ***
Soluble fertilizer	21.8 ± 1.40b	16.9 ± 0.85a	0.008 **	17.5 ± 1.1a	12.5 ± 0.41a	<0.001 ***
*p* value ^†^	0.025 *	n.s.		n.s.	0.044 *	

Asterisks indicate significant effect levels (* *p* < 0.05, ** *p* < 0.01, *** *p* < 0.001), n.s. not significant. ^†^ One-way ANOVA at 5%, ^‡^ two-sample *t*-test at 5%.

**Table 7:** Correlation matrices of the nutrient concentrations (g kg^−1^ of shoot DM) and shoot DM yield (g) produced in GM and MO soils.

	Glenmore (GM) Soil		Molesworth (MO) Soil	
	Shoot DM	S Content	Shoot DM	S Content
S content	−0.65 ***		−0.19 n.s.	
P content	−0.53 ***	−0.40 **	−0.11 n.s.	0.41 *

Asterisks indicate significance levels of the relations (* *p* < 0.05, ** *p* < 0.01, *** *p* < 0.001), n.s. not significant.

**Table 8:** Results of soil chemical and particle-size distribution before the establishment of the experiment.

Soil Analysis	Molesworth	Glenmore	By Method of
pH (H_2_O)	4.7	5.0	[68]
Olsen P (µg/mL)	13	18	[69]
Resin P (mg/kg)	24	31	[70]
P retention (%)	59	42	[68]
Inorganic P (mg/kg)	160	196	[71,72,73]
Organic P (mg/kg)	440	696	
P organic/P inorganic ratio	2.75	3.55	
Sulphate sulphur (µg/g)	9	15	[74]
Reserve K (me/100 g)	6.45	2.10	[75]
Anaerobic Min N (kg/ha)	102	169	[76]
Organic matter (% w/w)	8.5	10.6	[68]
Exchangeable Al (mg/kg)	21	8	[77]
Total N (% w/w)	0.38	0.53	(Dumas combustion method using an Elementar Vario Max Cube Analyzer)
Total C (% w/w)	4.91	6.18	
Carbon/Nitrogen	12.9	11.7	
CEC (meq/100 g)	14	17	[78]
Ca (meq/100 g)	0.9	4.7	[79]
Mg (meq/100 g)	0.43	0.79	
K (meq/100 g)	0.40	0.36	
Na (meq/100 g)	0.06	0.07	
Base saturation (%)	12.9	34.1	
Particle-Size distribution %			ISSS Classification
Clay (0.05–2 µm)	17	13	
Sand (20–2000 µm)	51	48	
Silt (2–20 µm)	32	40	

ISSS International Society of Soil Science.

**Table 9:** Chemical composition of the fertilizers used in the experiment.

		pH_w_	P	K	S	Ca	Mg	Na	Al	As	Cd
**Phosphogypsum**	% (wt/wt)	3.5	0.54	0.08	11.30	16.11	0.03	0.19	0.12	4.10^−4^	2.10^−4^
**Monocalcium phosphate**	% (wt/wt)	-	24.6	-	0.1	15.9	-	-	-	-	-
**Sodium sulphate**	% (wt/wt)	-	-	0.01	22.6	0.01	-	-	-	0	0

wt/wt = weight/weight.

## Supplementary Materials

The following are available online at https://www.mdpi.com/2223-7747/9/7/883/s1, Figure S1: Lime addition effect on overall average root dry matter (DM) accumulated under each soil (MO and GM indicate Molesworth and Glenmore soils, respectively). Error bars are standard errors (±SE, n = 28), Asterisks indicate the significance of two-sample *t*-test at 5% for liming (2 t ha^−1^) effect compared to no lime conditions (0 t ha^−1^) (* *p* < 0.05, ** *p* < 0.01, *** *p* < 0.001).
