# Supplementary material for: Impacts of Phosphogypsum, Soluble Fertilizer and Lime Amendment of Acid Soils on the Bioavailability of Phosphorus and Sulphur under Lucerne (Medicago sativa)
Source: Plants (Basel). 2020 Jul 13;9(7):883. doi: 10.3390/plants9070883 (PMC7411756; doi:10.3390/plants9070883)
Supplement: Supplementary file 1 [file plants-09-00883-s001.pdf]

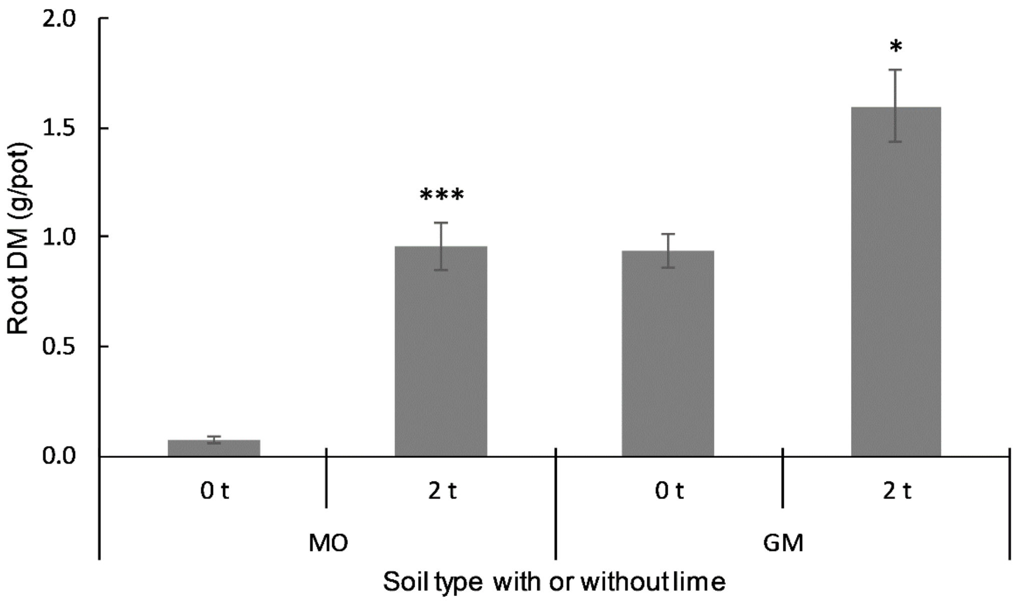


**Figure S1.** Lime addition effect on overall average root dry matter (DM) accumulated under each soil (MO and GM indicate Molesworth and Glenmore soils, respectively). Error bars are standard errors (± SE, n = 28), Asterisks indicate the significance of two-sample t-test at 5% for liming (2 t $ha^{-1}$) effect compared to no lime conditions (0 t $ha^{-1}$) (*$p < 0.05$, **$p < 0.01$, ***$p < 0.001$).
